# How Hahnemann Cured

**Published:** 1894-05

**Authors:** Wm. Bœricke

**Affiliations:** 834 Sutter Street, San Francisco, Cal.


### HOW HAHNEMANN CURED.

834 Sutter Street,
San Francisco, Cal., March 16th, 1894.

Editor Homœopathic Physician:—As a contribution to the question, "How Hahnemann Cured?" I inclose the following copy of the report of two cases sent by Hahnemann to Bœnninghausen. There are probably many of your readers who have not come across it. It is published in translation in the *North American Journal of Homœopathy*, November, 1879, and forms a part of a most interesting article by Bœnninghausen on "The Three Precautionary Rules of Hahnemann."

Yours respectfully,

Wm. Bœricke, M. D.

